# Combined in vivo MRI assessment of locus coeruleus and nucleus basalis of Meynert integrity in amnestic Alzheimer’s disease, suspected-LATE and frontotemporal dementia

**DOI:** 10.1186/s13195-024-01466-z

**Published:** 2024-05-03

**Authors:** Julien Lagarde, Pauline Olivieri, Matteo Tonietto, Camille Noiray, Stéphane Lehericy, Romain Valabrègue, Fabien Caillé, Philippe Gervais, Martin Moussion, Michel Bottlaender, Marie Sarazin

**Affiliations:** 1 Department of Neurology of Memory and Language, GHU Paris Psychiatry and Neurosciences, Hôpital Sainte Anne, Paris, France; 2 Université Paris-Saclay, Service Hospitalier Frédéric Joliot CEA, CNRS, Inserm, BioMaps, Orsay, F- 91401 France; 3 Université Paris-Cité, Paris, France; 4 Centre de NeuroImagerie de Recherche - CENIR, Institut du Cerveau et de la Moelle épinière - ICM, Paris, F-75013 France; 5 Sorbonne Université, UPMC Univ Paris 06, UMR S 1127, Inserm U 1127, CNRS UMR 7225, ICM, Paris, F-75013 France; 6 Centre d’Evaluation Troubles Psychiques et Vieillissement, GHU Paris Psychiatrie & Neurosciences, Hôpital Sainte Anne, Paris, F-75014 France; 7 UNIACT, Neurospin, Gif-sur-Yvette, CEA F-91191 France

**Keywords:** Locus coeruleus, Nucleus basalis of Meynert, Alzheimer, LATE, FTD, MRI

## Abstract

**Background:**

The locus coeruleus (LC) and the nucleus basalis of Meynert (NBM) are altered in early stages of Alzheimer’s disease (AD). Little is known about LC and NBM alteration in limbic-predominant age-related TDP-43 encephalopathy (LATE) and frontotemporal dementia (FTD). The aim of the present study is to investigate in vivo LC and NBM integrity in patients with suspected-LATE, early-amnestic AD and FTD in comparison with controls.

**Methods:**

Seventy-two participants (23 early amnestic-AD patients, 17 suspected-LATE, 17 FTD patients, defined by a clinical-biological diagnosis reinforced by amyloid and tau PET imaging, and 15 controls) underwent neuropsychological assessment and 3T brain MRI. We analyzed the locus coeruleus signal intensity (LC-I) and the NBM volume as well as their relation with cognition and with medial temporal/cortical atrophy.

**Results:**

We found significantly lower LC-I and NBM volume in amnestic-AD and suspected-LATE in comparison with controls. In FTD, we also observed lower NBM volume but a slightly less marked alteration of the LC-I, independently of the temporal or frontal phenotype. NBM volume was correlated with the global cognitive efficiency in AD patients. Strong correlations were found between NBM volume and that of medial temporal structures, particularly the amygdala in both AD and FTD patients.

**Conclusions:**

The alteration of LC and NBM in amnestic-AD, presumed-LATE and FTD suggests a common vulnerability of these structures to different proteinopathies. Targeting the noradrenergic and cholinergic systems could be effective therapeutic strategies in LATE and FTD.

## Background

There is currently growing interest in the involvement of neuromodulatory subcortical systems in Alzheimer’s disease (AD) and, more broadly, in neurodegenerative pathologies [1]. The locus coeruleus (LC) and the nucleus basalis of Meynert (NBM) are the main nuclei of two important neuromodulatory systems, the noradrenergic and cholinergic systems respectively. The LC is the major source of noradrenaline in the brain [2] and plays a critical role in several brain functions, including cognition, sleep, emotion, and stress responses [3, 4]. The NBM is a major cholinergic hub targeting limbic and cortical areas, and is involved in cortical activation, attention, executive functions, and memory processes [5, 6].

Autopsy studies have revealed the degeneration of LC neurons in prodromal AD, associated with disease severity [7–9]. They also mention a reduction in cortical choline-acetyl transferase activity [10] related to neuronal loss in the basal forebrain, mainly in the Ch4 neuronal group constituting the NBM, in the early and predementia stages of the disease [11–14]. Neuropathological studies have also shown that the LC and NBM are among the earliest sites of detectable tau pathology in AD [14, 15], and some authors suggest that tau pathology in the LC precedes the onset of first symptoms by several decades [16, 17] and may underlie the development of aberrant protein aggregates in interconnected brain regions [18, 19].

In recent years, new MR imaging techniques have emerged to measure the LC signal intensity (LC-I), which likely reflects LC neuronal integrity [20]. In vivo NBM analysis has also been made possible by the development of atlases derived from combined post mortem MRI and histology [21, 22]. Several MRI volumetric studies have shown consistent NBM atrophy in typical AD from the early stage [23–25].

Limbic-predominant age-related TDP-43 encephalopathy (LATE) is a recently described entity characterized by a progressive amnestic syndrome mimicking early AD, but not due to AD pathology. LATE neuropathological changes (LATE-NC) are characterized by abnormal neuronal deposits of TDP-43 protein localized mainly in limbic areas with or without coexisting hippocampal sclerosis (HS) [26–28]. Little is known about NBM and LC alteration in LATE patients. A recent update of the LATE-NC definition based on published data does not mention these structures [27]. In vivo MRI studies showed a reduction in basal forebrain volume in two small groups of autopsy-confirmed limbic TDP-43 cases at different stages of the disease (asymptomatic to dementia) [29, 30]. TDP-43 pathology can also be observed in other neurodegenerative diseases, such as frontotemporal dementia (FTD-TDP), in which tau pathology represents the other main proteinopathy involved (FTD-tau). Recent studies suggested LC neuronal loss [31, 32] and a reduction in NBM volume in FTD, possibly more pronounced in FTD-tau than in FTD-TDP [33].

Because AD and LATE share a similar initial amnestic clinical phenotype despite distinct proteinopathies, studying both NBM and LC in early amnestic AD and presumed-LATE patients could provide new information on the initial target of proteinopathies and could open up new avenues for therapeutic strategies.

The aim of the present study was to (a) investigate the LC and NBM integrity in patients with identical clinical phenotypes underpinned by distinct mechanisms (TDP-43 proteinopathy in presumed-LATE and amyloid/tau pathology in amnestic AD) by combining in vivo MRI assessment of LC-I and NBM volume, (b) explore in a supplementary analysis patients with another type of TDP-43 or tau pathology (FTD) and (c) analyze the relation between LC-I/NBM volume and cognition as well as medial temporal and cortical atrophy in these groups of patients. We expected to find alterations in both NBM and LC in each disease to varying degrees.

## Methods

### Patients

The 72 participants in this study were recruited as part of the prospective Shatau7-Imatau protocol (NCT02576821-EudraCT2015-000257-20). The Ethics Committee (Comité de Protection des Personnes Ile-de-France VI) approved the study (Protocole n° 13–15). All subjects provided written informed consent.

Patients with amnestic AD at the MCI or mild dementia stage (*n* = 23) were included according to the following criteria: (i) amnestic syndrome of the hippocampal type; (ii) pathophysiological markers suggestive of AD, defined by CSF AD profile and both positive amyloid ([^11^C]-PiB) and tau ([^18^F]-flortaucipir) PET imaging; (iii) clinical dementia rating (CDR) scale ≤ 1.

Patients with presumed-LATE (*n* = 17) were included according to the following criteria: (i) progressive amnestic syndrome of the hippocampal type; (ii) CSF biomarkers not suggestive of AD; (iii) negative amyloid and/or tau PET imaging, as previously described [34]; (iv) clinical dementia rating (CDR) scale ≤ 1; (v) no extrapyramidal or neurological signs suggestive of other diseases, such as Parkinson’s disease, progressive supranuclear palsy, corticobasal degeneration, fronto-temporal dementia, Lewy body disease, epilepsy; (vi) no sleep apnea; (vii) no vascular lesion on MRI that could account for amnesia; (viii) no argument in favor of a neurological diagnosis other than presumed-LATE after two years of follow-up, including a second negative tau-PET imaging when available (*n* = 11/17). Note that 3 patients had slightly positive amyloid PET imaging (global cortical index- GCI- SUVr between 1.45 and 1.87, whereas it is always greater than 2 in our AD patients) despite normal CSF biomarkers and negative tau-PET imaging. They were considered as presumed-LATE patients with amyloid copathology. To facilitate reading, we will refer to presumed-LATE as LATE in the rest of this article [28].

Patients with FTD (*n* = 17) were included according to the following criteria: (i) behavioral modifications fulfilling the criteria for bvFTD [35] or language impairment fulfilling the criteria for semantic primary progressive aphasia (temporal FTD) [36]; (ii) characteristic findings on neuroimaging ([^18^F]-FDG-PET or SPECT); (iii) pathophysiological markers not suggestive of AD, defined by negative amyloid PET and/or CSF biomarkers (PET imaging was not performed in 3 FTD patients). Among our FTD patients, one carried a *GRN* mutation and another a *MAPT* mutation. Five FTD patients had a predominantly temporal variant, and the others a predominantly frontal variant.

Fifteen healthy elderly controls were included according to the following criteria: (i) Mini-Mental State Examination (MMSE) score ≥ 27/30; (ii) normal neuropsychological assessment; (iii) CDR = 0; (iv) no memory complaints; (v) negative amyloid PET imaging ( – GCI < 1.4).

We did not include subjects with (i) systemic illnesses that could interfere with cognitive functioning; (ii) Fazekas score > 2.

Blood samples were drawn to determine *APOE* and *c9orf72* genotypes as well as plasma progranulin levels in patients.

### Functional and cognitive assessment

All participants underwent a standardized neurological and neuropsychological examination including the MMSE, the Mattis Dementia Rating scale (Mattis DRS), CDR scale and a standardized cognitive battery assessing verbal episodic memory, executive functions, gesture praxis, visuo-constructive functions and language. As previously described [37], we defined a verbal episodic memory score, a parietal cognitive score and an executive cognitive score.

### Magnetic resonance imaging

All subjects underwent magnetic resonance imaging at the Centre de Neuro-Imagerie de Recherche (CENIR, ICM, Paris) using a 3T whole-body PRISMA 64-channel system (Siemens). The MRI examination included (a) a three-dimensional (3D) T1-weighted volumetric magnetization-prepared rapid gradient echo (MP-RAGE) sequence (repetition time/echo time/flip angle: 2300 ms/3.43 ms/9°, inversion time = 900 ms, and voxel size: 1 × 1 × 1 mm^3^); (b) two-dimensional axial turbo spin echo T1-weighted images (repetition time/echo time/flip angle: 900ms/15 ms/180°, three averages, voxel size: 0.4 × 0.4 × 3mm3, acquisition time: 7 min) that were positioned on the sagittal 3D T1-weighted images perpendicular to the posterior border of the brainstem from the lower part of the pons to the upper part of the midbrain, covering the entire LC; (c) a Fluid Attenuated Inversion Recovery (FLAIR) sequence.

### Determination of LC signal intensity (LC-I)

**Fig. 1:**
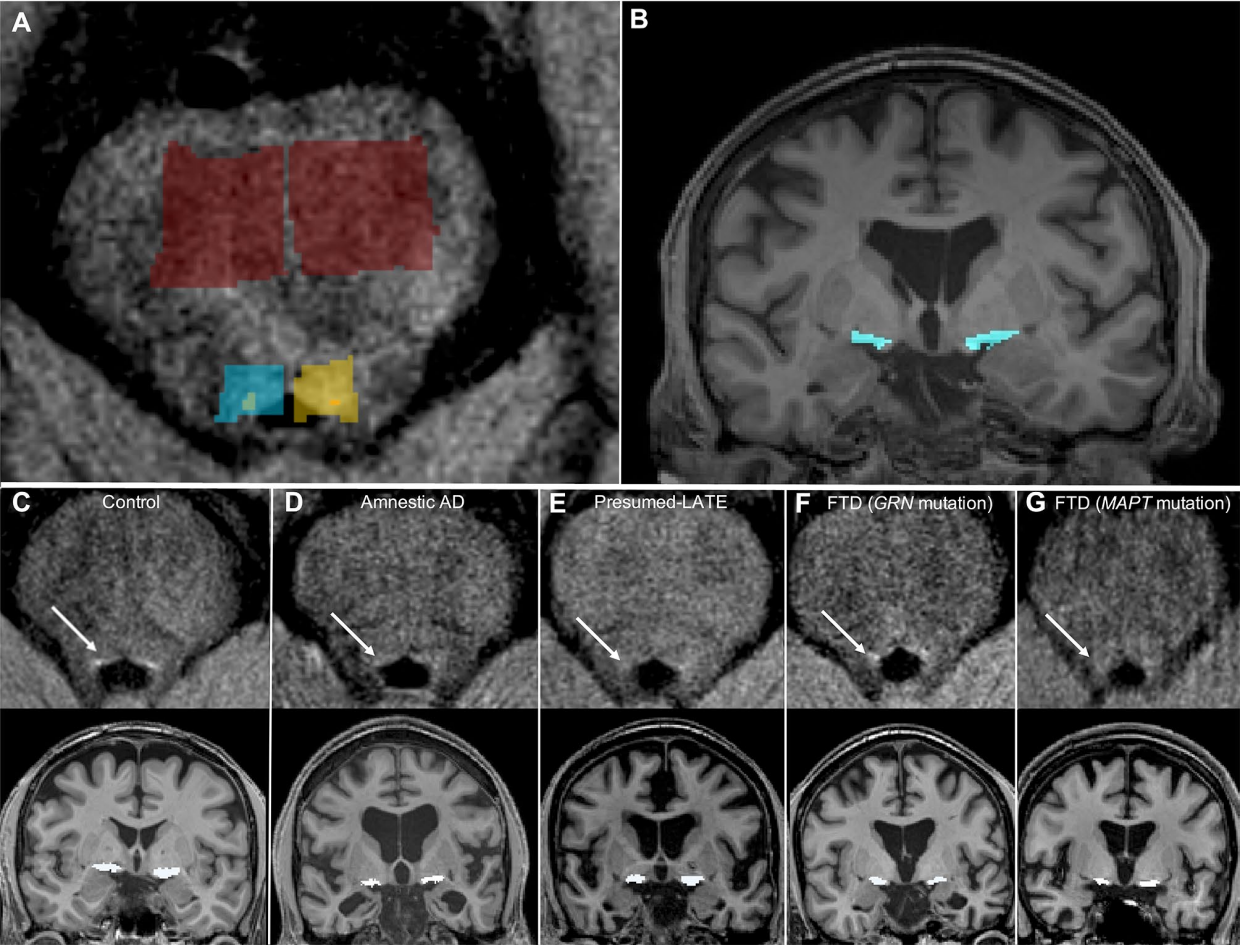
Visualization of LC-I and NBM volume. Positioning of the boxes used for LC-I assessment (rostral pontomesencephalic area used as a reference region in red, and bilateral regions containing the coeruleus-subcoeruleus complexes in blue and yellow) **(A)**, and of the mask for NBM volume measurement **(B)**. Representative images of the LC-I (top row) and NBM volume (bottom row) in one control subject **(C)**, one AD patient **(D)**, one amyloid-negative suspected-LATE patient **(E)**, and the two FTD patients carrying the *GRN***(F)** and *MAPT***(G)** mutations

The analysis was performed using a previously described automated method [38–40]. Three regions (serving as bounding boxes) were manually defined on the International Consortium for Brain Mapping template and were resampled onto the individual turbo spin echo T1-weighted images. The first region corresponded to the rostral pontomesencephalic area (6200 mm^3^) and was used as a reference region for signal normalization between subjects. This normalization allowed the direct comparison of intensity values between subjects. The other regions corresponded to the bilateral regions containing the coeruleus-subcoeruleus complexes (700 mm^3^ each), avoiding any other structure that could be considered as ‘bright’ such as the substantia nigra. We used an in-house software to automatically determine the 10 brightest connected voxels bilaterally, which were considered as the LC areas (Fig. 1) [38]. The appropriate location of voxels in the LC area was checked visually on each image (none was modified). Due to technical issues, LC-I values were missing in one LATE patient and in 5 FTD patients.

### Nucleus basalis of Meynert

All 3D-T1 images were corrected for field bias using the N4 algorithm implemented in Advanced Normalization Tools (ANTs) 2.2.0 [41]. A brain mask was automatically segmented using the volBrain pipeline [42] and manual corrections were performed by a neurologist (PO) when necessary. The skull-stripped 3D-T1 images were then normalized to the Colin 27 Average Brain included in the JuBrain Anatomy Toolbox [43] using a nonlinear deformation calculated with ANTs. This transformation was subsequently inverted and applied to the NBM mask included in the JuBrain Anatomy Toolbox to bring the mask from the Colin 27 Average Brain space to the 3D-T1 native space (Fig. 1). From this mask, the volume of the NBM was calculated (average of left and right sides) and normalized to the intracranial volume (ICV).

### Cortical VOIs

MRIs were also processed with the FreeSurfer 6.0.0 processing stream (http://surfer.nmr.mgh.harvard.edu/). We visually inspected the FreeSurfer parcellation results to identify segmentation abnormalities and performed manual edits when necessary. We studied grey matter volumes in the following specific regions of interest: (i) the hippocampi and entorhinal cortices, whose atrophy is associated with amnestic syndromes (ii) the amygdala, which are affected in non-AD pathologies, especially in LATE [13]. We also considered the cortical thickness (CT) in the following VOIs: (i) a temporal meta-VOI composed of the entorhinal cortex, parahippocampus, fusiform gyrus, inferior and middle temporal cortex [44]; (ii) a lateral temporal VOI; (iii) a lateral parietal VOI; (iv) a medial parietal VOI; and (v) a frontal VOI (as defined in Ossenkoppele et al.) [45]. We considered the average values of the left and right sides.

### Statistical analysis

Data were analyzed using R version 3.6.1 (R Core Team, 2017). Differences between subject groups were assessed using analysis of covariance (ANCOVA). Age and sex were included as covariates.

The correlation analyses between LC-I or NBM volume and the clinical variables or the medial temporal volume/CT in the VOIs in patients were performed by using linear partial correlation analyses, which were corrected for the diagnostic group (for the correlations within the whole patient group), age and sex (the latter for imaging variables).

The Bonferroni correction for multiple comparisons was used (for 6 tests in the comparison of LC-I and NBM volume between groups, for 48 tests in the comparison of other imaging parameters, for 30 tests in the comparison of neuropsychological data between groups, for 48 tests in the correlation analyses with neuropsychological data and for 64 tests in the correlation analyses with imaging data). The level of statistical significance was set at *p* < 0.05.

## Results

### Characteristics of the participants

The main demographic, cognitive and imaging data for the control, AD, LATE and FTD groups are summarized in Table 1. LATE patients were older than other patient groups. AD and LATE patients had similar cognitive phenotypes. FTD patients tended to be more significantly impaired than AD and LATE patients in all cognitive domains, the difference being significant for the parietal and the executive scores. Medial temporal volumes and CT were lower in all regions in all patient groups compared to controls. Compared to the other patient groups, CT in the temporal and frontal lobes was lower in the FTD group.

**Table 1:** Main demographic, clinical, and imaging characteristics (mean (SD))

		Controls (*n* = 15)	AD (*n* = 23)	LATE (*n* = 17)	FTD (*n* = 17)
Age (years)		68.7 (3.6)	70.4 (6.5)	77.3 (5.2)^#^	70.1 (8.6)
Sex (M/F)		5/10	12/11	11/6	9/8
*APOE* genotype (n with at least one E4 allele)		2	16	2	4
Medication	AChEI (n)	NA	16	2	1
	SSRI (n)	NA	12	7	10
	SNRI (n)	NA	4	0	1
MMSE (/30)		28.8 (1)	24.4 (3.1)*	24.6 (2.8)*	20.9 (5.2)*
Mattis DRS (/144)		141 (3.2)	126 (7.6)*	129 (7.4)	110 (25.5)*
Memory score (FCSRT 96)		80.7 (5.2)	32.7 (16.1)*	38.1 (16.3)*	38.2 (25.6)*
Parietal score (/188)		185.6 (1.8)	180 (5.4)	182.5 (3)	149.6 (31.3)^#^
Executive score		61.6 (8.8)	47.7 (9.7)*	48.2 (9.2)*	30.3 (14.6)^#^
Hippocampal Volume		2.45 (0.24)	1.92 (0.3)*	1.72 (0.35)*	1.77 (0.36)*
Amygdala volume		0.96 (0.11)	0.75 (0.11)*	0.72 (0.16)*	0.68 (0.23)*
Entorhinal volume		1.28 (0.2)	0.93 (0.31)*	0.9 (0.26)*	0.85 (0.3)*
Temporal meta-VOI CT (mm)		2.86 (0.12)	2.53 (0.2)*	2.55 (0.22)*	2.25 (0.3)^#^
Lateral temporal VOI CT (mm)		2.78 (0.13)	2.5 (0.14)*	2.48 (0.22)*	2.29 (0.23)^#^
Lateral parietal VOI CT (mm)		2.33 (0.15)	2.14 (0.17)*	2.15 (0.15)*	2.07 (0.15)*
Medial parietal VOI CT (mm)		2.31 (0.13)	2.13 (0.16)*	2.12 (0.11)*	1.99 (0.19)^#^
Frontal VOI CT (mm)		2.57 (0.1)	2.39 (0.15)*	2.32 (0.13)*	2.19 (0.19)^#^

AD: Alzheimer’s disease, LATE: Limbic-predominant age-related TDP-43 encephalopathy, FTD: frontotemporal dementia, AChEI: acetylcholinesterase inhibitors, SSRI: selective serotonin reuptake inhibitors, SNRI: serotonin-norepinephrine reuptake inhibitors, MMSE: Mini-Mental State Examination, Mattis DRS: Mattis Dementia Rating Scale, FCSRT: Free and Cued Selective Reminding Test, VOI: Volume Of Interest, CT: Cortical Thickness, mm: millimetres

^*^p < 0.05 in the comparison with controls after Bonferroni correction for 30 tests for neuropsychological data and for 48 tests for the imaging data

# *p* < 0.05 in the comparison with the other groups after Bonferroni correction for 30 tests for neuropsychological data and for 48 tests for the imaging data

### Locus coeruleus signal intensity (LC-I)

**Fig. 2:**
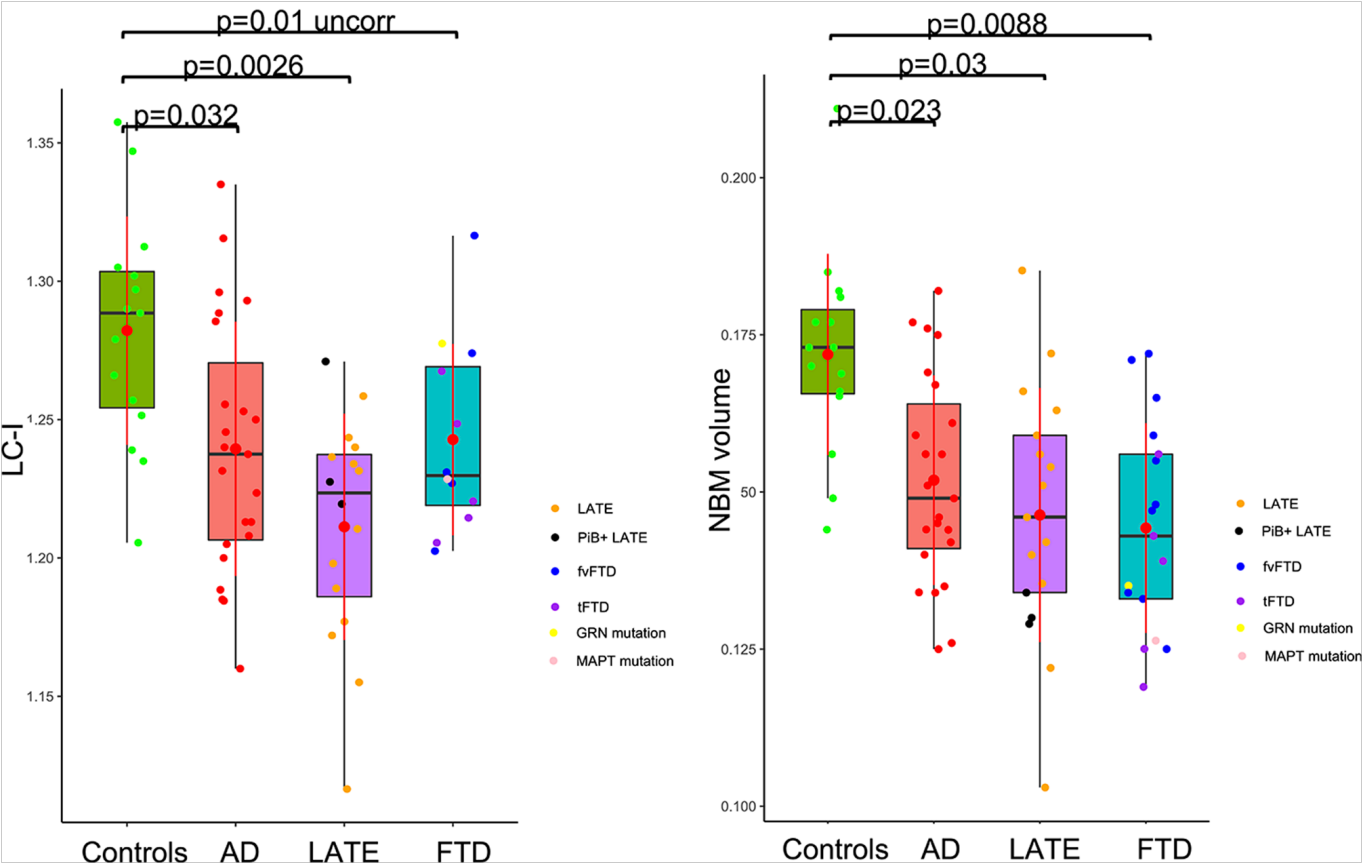
Boxplots representing the locus coeruleus signal intensity and NBM volume across groups. The boxes indicate the median and the upper and lower quartiles. The red point and line on each boxplot indicate the mean value and the standard deviation. AD = Alzheimer’s disease, LATE = limbic-predominant age-related TDP-43 encephalopathy, FTD = Frontotemporal dementia (fv = frontal variant, t = temporal variant), PiB + LATE = amyloid-positive LATE

The LC-I was significantly lower in the three patient groups compared with controls. The difference did not survive correction for multiple comparisons in the FTD group (Fig. 2). We did not observe a distinctive pattern within the FTD group in patients with a temporal variant (most often underpinned by TDP-43 pathology) or with a frontal variant. The patient with the *MAPT* mutation had a rather lower LC-I than the *GRN* mutation carrier, but it is difficult to draw conclusions from isolated observations.

### NBM volume

NBM volume was significantly lower in the three patient groups compared with controls (Fig. 2). As with LC-I, we did not observe a distinctive pattern within the FTD group in patients with temporal or frontal variants. The *MAPT* mutation carrier had a slightly lower NBM volume than the *GRN* mutation carrier (Fig. 2).

### Correlations between LC-I or NBM volume and cognitive scores

We found no significant correlation between LC-I or NBM volume and cognitive variables when considering all patients. When performing the analysis within each subgroup, we found only a significant correlation between NBM volume and Mattis DRS score in AD patients, which did not resist correction for multiple comparisons (Table 2).

**Table 2:** Correlations between LC-I/NBM volume and the cognitive variables and medial temporal/cortical atrophy (r^2^ values)

		MMSE	Mattis DRS	Memory score	Parietal score	Executive score	HV	Amyg vol	EC vol	Temporal meta-VOI CT	Lateral temporal VOI CT	Lateral parietal VOI CT	Medial parietal VOI CT	Frontal VOI CT
All patients	LC-I	0.15	0.14	0.14	0.14	0.14	0.14	0.17	0.16	0.18	0.2	0.19	0.15	0.17
	NBM	0.08	0.05	0.09	0.06	0.05	**0.49**	**0.5**	**0.36**	0.24*	0.17*	0.13	0.16*	0.16*
AD	LC-I	0.11	0.15	0.12	0.13	0.03	0.13	0.29*	0.18	0.23*	0.29*	0.12	0.11	0.13
	NBM	0.15	0.27*	0.08	0.07	0.06	0.48*	**0.6**	0.5*	0.38*	0.29	0.28	0.36*	0.41*
LATE	LC-I	0.05	0.18	0.13	0.27	0.11	0.27	0.07	0.06	0.13	0.23	0.34	0.06	0.2
	NBM	0.19	0.1	0.07	0.07	0.22	0.46*	0.43*	0.44*	0.2	0.16	0.09	0.22	0.24
FTD	LC-I	0.05	0.08	0.09	0.13	0.07	0.2	0.06	0.06	0.08	0.1	0.33	0.13	0.08
	NBM	0.03	0.03	0.38	0.1	0.03	0.6*	**0.65**	0.32	0.42*	0.34	0.28	0.28	0.23

AD: Alzheimer’s disease, LATE: Limbic-predominant age-related TDP-43 encephalopathy, FTD: frontotemporal dementia, MMSE: Mini-Mental State Examination, Mattis DRS: Mattis Dementia Rating Scale, HV: hippocampal volume, Amyg vol: amygdala volume, EC vol: entorhinal cortex volume, VOI: Volume Of Interest, CT: Cortical Thickness

Values in bold are significant after Bonferroni correction for 48 tests for the neuropsychological data and for 64 tests for the imaging data

^*^p < 0.05 uncorrected

### Correlations between LC-I or NBM volume and medial temporal volume /cortical thickness

When considering all patients, we found significant correlations between NBM volume and hippocampal volume (*p* = 0.0000012), amygdala volume (*p* = 0.0000014) and entorhinal volume (*p* = 0.00063). We also found a correlation between NBM volume and the CT in the temporal meta-VOI, and to a lesser extent in the lateral temporal, medial parietal and frontal VOIs, which did not survive correction for multiple comparisons (*p* = 0.0017, 0.02, 0.033 and 0.037, uncorrected). We did not find any significant result for the LC-I.

In the AD subgroup, we found a significant correlation between NBM volume and amygdala volume (*p* = 0.017). We also found correlations between NBM volume and hippocampal and entorhinal volumes, as well as to a lesser extent with the CT in the temporal meta-VOI, medial parietal and frontal VOIs, which did not survive correction for multiple comparisons (*p* = 0.0034, 0.002, 0.018, 0.027 and 0.013 uncorrected, respectively). We also found correlations between LC-I and amygdala volume, as well as the CT in the lateral temporal VOI and in the temporal meta-VOIs, which did not survive correction for multiple comparisons (*p* = 0.0018, 0.0047 and 0.019 uncorrected, respectively).

In the LATE group, we found correlations between NBM volume and hippocampal, amygdala and entorhinal volumes, which did not survive correction for multiple comparisons (*p* = 0.0095, 0.013, 0.012, uncorrected, respectively).

**Fig. 3:**
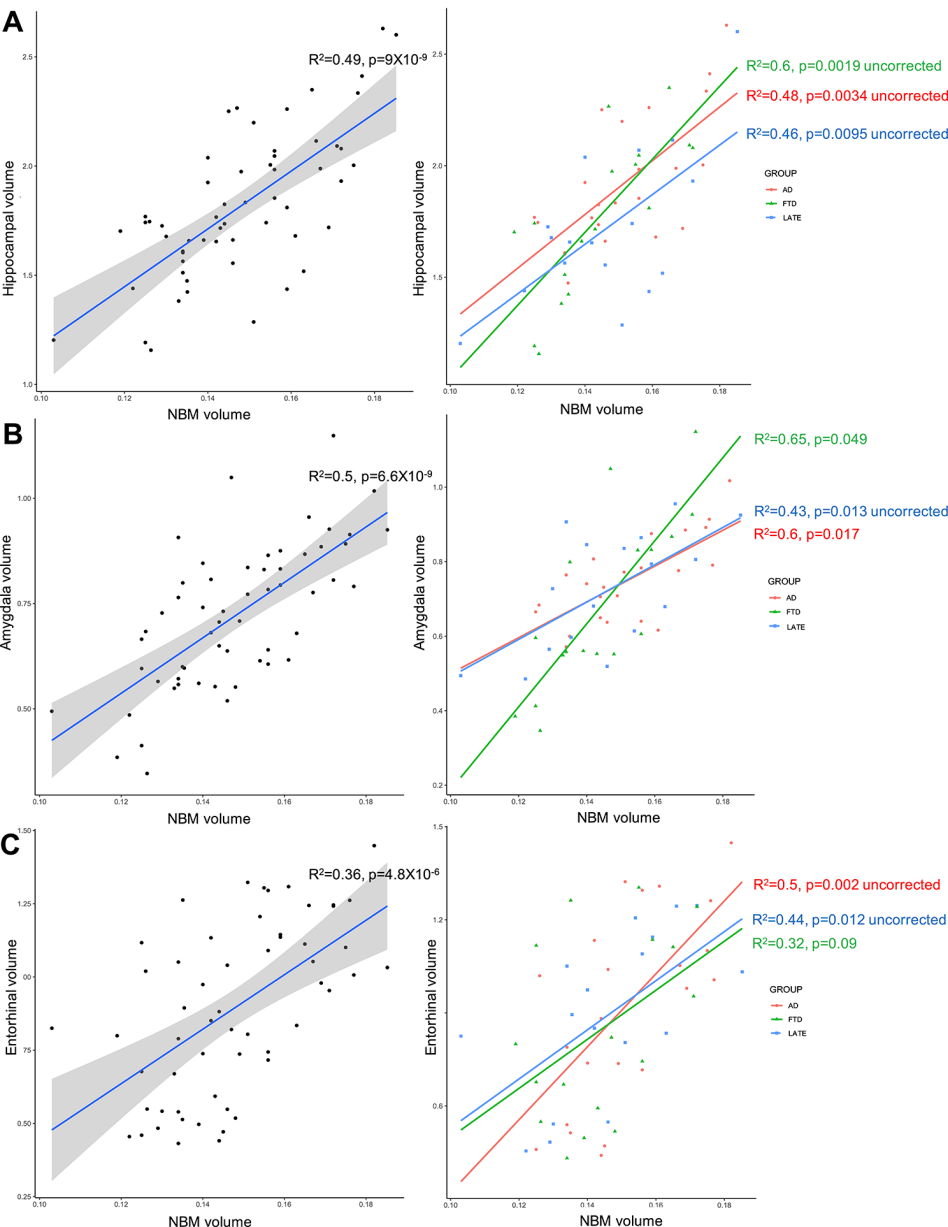
Correlation between the NBM volume and the hippocampal **(A)**, amygdala **(B)** and entorhinal **(C)** volumes in the whole patient group (left panels) and in the subgroups (right panels)

In the FTD subgroup, we found a significant correlation between NBM volume and amygdala volume (*p* = 0.049). We also found a correlation between NBM volume and hippocampal volume, as well as the CT in the temporal meta-VOI, which did not survive correction for multiple comparisons (*p* = 0.0019 and 0.028, uncorrected) (Table 2; Fig. 3).

## Discussion

In this study, we report, for the first time to our knowledge, the results of a combined in vivo MRI assessment of the degree of LC and NBM alteration in presumed-LATE and early amnestic-AD patients. One strength of this study is that the patient groups were very precisely defined in terms of clinical phenotype and relevant pathophysiological biomarkers, including CSF AD biomarkers, amyloid and tau PET imaging, as well as a 2-year clinical follow-up. As these two diseases have a similar cognitive phenotype and pattern of medial temporal atrophy, but are underpinned by different proteinopathies, they provide a good model for analyzing whether alterations in NBM and LC-I differ depending on the pathophysiological process. The main finding is that the LC and NBM were significantly impaired in the AD and LATE groups, with NBM volume particularly correlated with that of the amygdala in AD. In addition, we included patients with temporal variant FTD, in whom TDP-43 pathology is highly suspected, and with frontal variant FTD, which may be underpinned by TDP-43 or tau pathologies. We found lowered NBM volume and a slightly less marked alteration in LC-I, independently of the clinical phenotype in these patients.

In early amnestic-AD patients, our results are in accordance with both neuropathological and MRI data showing early involvement of the NBM [46, 47] and of the LC [40, 48], both structures being affected by tau pathology at an early stage of the disease [14–16]. Recent studies also showed associations between LC-I and early tau accumulation in the entorhinal cortex [49], and between NBM volume and neurodegeneration in the entorhinal and perirhinal cortices [50], which argues in favour of an early involvement of these subcortical structures in AD pathogenesis, particularly as a potential starting point for the spread of proteinopathies.

In LATE patients, we also found an alteration of both LC-I and NBM volume in comparison with the controls. These results do not seem to be influenced by a possible amyloid copathology. LC alteration has never been specifically studied in non-AD amnesia, apart from a recent study in amnestic MCI that did not use pathophysiological biomarkers to clarify the etiological diagnosis [51]. Our results suggest that LC could be an early target of proteinopathies, not only for tau but also for TDP-43 pathology. Few studies have investigated NBM alteration in LATE. One neuropathological study reported TDP-43 deposition in the basal forebrain of patients with hippocampal sclerosis-ageing in the absence of AD copathology, suggesting a direct vulnerability of NBM to TDP-43 pathology [52]. Lowered NBM volume measured by antemortem MRI was recently reported in autopsy-confirmed LATE patients [29, 30]. Our study reinforces these results in a group of suspected-LATE patients at an early symptomatic stage.

In FTD, despite the clinical and probable neuropathological heterogeneity of our group, we observed significantly lower LC-I and NBM volume in comparison with controls, with no obvious influence of semantic or behavioral clinical phenotype. Previous data reported a loss of LC neurons in FTD, predominating in FTD-tau versus FTD-TDP [31, 32], as well as lowered NBM volume particularly pronounced in FTD-tau [33, 46]. Due to the small size of our group, it is not possible to conclude that there is any distinction between temporal (suspected TDP-43 pathology) and frontal (TDP-43 or tau pathology) variants. Further studies involving a larger group of patients with temporal FTD will also be needed to better define the alteration of LC and NBM in this TDP-43 proteinopathy and to compare it with that observed in LATE. A recent study using diffusion MRI also found microstructural alterations of the pathway connecting the LC to the transentorhinal cortex in both AD and bvFTD, which were even more pronounced in the latter [53].

The lack of correlation between LC-I and cognition in AD and LATE is surprising, given the expected role of the noradrenergic system in attention and memory. This could be related to the small sample size and to different biases, in particular to the measurement method of the LC-I that indirectly reflects neuronal loss in a very restricted part of the LC and probably takes less account of the heterogeneity of the organization of the LC neurons. Another potential explanation of the lack of correlation between LC-I and cognition in AD could be the relative homogeneity of this group in terms of pathophysiological biomarkers. This could complicate the identification of the association between LC disruption and cognitive impairment, which is likely to be mediated by AD pathology [48] and could be more noticeable in more heterogeneous cohorts.

Regarding NBM volume, we found correlations with global cognitive efficiency (Mattis DRS) in AD patients, consistent with previous studies [46, 54], but not in LATE. It may reflect different implications of altered NBM in cognition in each condition, but could also be influenced by cholinergic treatment in AD patients.

We found significant correlations between the NBM volume and that of the medial temporal structures, such as the amygdala, hippocampus and entorhinal cortex, the strongest correlation being observed with the amygdala. This is in accordance with a previous study that reported an association between NBM and amygdala volumes in amnestic MCI included on the basis of clinical criteria alone [55]. This particular relationship between NBM and amygdala volumes does not seem fortuitous, because in AD, the neuronal loss in Ch4 predominates in its anterolateral and posterior parts, which are those emitting projections towards the amygdala and the temporal lobe [46, 56]. It is also in accordance with the pattern of tau propagation defined in Braak’s staging, which first mentions the entorhinal cortex and amygdala before extending to cortical areas, as well as with the stages of propagation of the abnormal TDP-43 protein in LATE, starting in the amygdala (stage 1), then in the hippocampus (stage 2) and finally reaching the middle frontal gyrus (stage 3) [27]. Due to the heterogeneity of the FTD group, it seems more difficult to interpret correlation analyses in these patients, but we also found a significant result between NBM and amygdala volumes.

The results obtained here highlight the potential interest of targeting not only the cholinergic system, but also the noradrenergic system in AD and in suspected LATE/TDP-43 pathology. This could be true both for symptomatic purposes (activation of β2-adrenoreceptors strengthens long-term potentiation and synaptic activity, thus improving learning and memory [57]), but also possibly to influence disease progression, given the relationship described between these neuromodulatory systems and certain components of the pathophysiology of these diseases, such as tau hyperphosphorylation (which is activated by the binding of Aß oligomers to α2-adrenoreceptors [58]) and neuroinflammation [57, 59]. β2-adrenoreceptor agonists may have beneficial effects on several neurodegenerative diseases and play a neuroprotective role [57]. Similarly, it has been suggested that acetylcholine has anti-inflammatory and neuroprotective properties in a number of neurodegenerative disorders. The alteration of NBM and LC integrity in suspected LATE argues for clinical trials evaluating the effects of cholinergic/noradrenergic treatments in progressive amnesia due to limbic TDP-43 pathology, for which no therapeutic option is currently recommended. The potential therapeutic targeting of cholinergic/noradrenergic systems in FTD merits further exploration.

### Limitations

The present study has several limitations. First, the relatively limited sample size probably limits statistical power, especially in heterogeneous groups such as FTD. The lack of neuropathological diagnosis is also a limitation, despite the use of combined pathophysiological markers of AD and a 2-year clinical follow-up. The methods used to explore LC and NBM alteration were not quite comparable. NBM assessment is based on the determination of a volume corresponding to the basal forebrain cholinergic cell group called Ch4 [56] and is normalized to the ICV, while LC integrity assessment is based on the determination of signal intensity in a limited number of LC voxels. This difference in the essence of the imaging data used could play a role in the correlation analyses and explain the absence of significant results with the LC-I, particularly for cognitive parameters. This could be addressed by automated LC segmentation methods under development, which seem more feasible and reproducible than manual segmentation, and probably more accurate than segmentation in standardized space with an LC ROI mask [60]. In addition, the methods used here did not allow us to explore the sub-regions of these two nuclei in more detail. Finally, they also fall short of exploring the noradrenergic and cholinergic systems in detail and should probably be combined with other tools targeting the determination of neurotransmitter concentration in the brain, the measure of LC and NBM activity [61], the functioning of noradrenergic/cholinergic receptors, and the interactions between the neurotransmitter systems [62].

## Conclusions

The present study showed LC and NBM alterations in both early amnestic-AD and LATE, correlating with medial temporal volume in areas described as affected in the early stages of the spread of tau or TDP-43 proteinopathies. These results underline that these structures could be common brain regions of vulnerability to different proteinopathies. The results observed in FTD also support this hypothesis, although it merits further exploration. The present study also suggests that targeting the noradrenergic and cholinergic systems could be effective therapeutic strategies in AD and LATE, the latter of which has no specific symptomatic treatment to date.

## Data Availability

The datasets used and/or analyzed during the current study are available from the corresponding author on reasonable request.

## Abbreviations

AD: Alzheimer’s disease
LATE: Limbic-predominant age-related TDP-43 encephalopathy
FTD: frontotemporal dementia
LC: locus coeruleus
LC-I: locus coeruleus signal intensity
NBM: nucleus basalis of Meynert
PET: Positron emission tomography
GCI: global cortical index
SUVr: standardized uptake value ratio
CSF: cerebrospinal fluid
CDR: Clinical Dementia Rating
MMSE: Mini-Mental State Examination
MRI: magnetic resonance imaging
VOI: volume of interest
CT: cortical thickness
ANTs: Advanced Normalization Tools
